# Citizen participation in the police domain: The role of citizens’ attitude and morality

**DOI:** 10.1002/jcop.21972

**Published:** 2018-03-25

**Authors:** Wendy Schreurs, José H. Kerstholt, Peter W. de Vries, Ellen Giebels

**Affiliations:** ^1^ University of Twente; ^2^ Toegepast Natuurwetenschappelijk Onderzoek (TNO)

## Abstract

Although there is a large potential of citizen capital in fighting crime and creating safer neighborhoods, in reality, only a small fraction of citizens is actively participating. This study examines the relationship between different types of actual participation behavior in the police domain from a citizen's stance and 3 different but interconnected psychological drivers: the attitude toward citizen participation, moral values, and moral emotions. A total of 217 Dutch citizens filled out an online questionnaire, assessing these drivers and the actual participatory actions they engaged in over the past year. The results show that 4 broad categories of participation behavior can be distinguished: social control (e.g., correcting others regarding their behavior); responsive participation (e.g., calling the police); collaborative participation (e.g., meeting with a police officer); and detection (e.g., joining a neighborhood watch). As expected, moral values had an indirect influence on participation via attitude and moral emotions. The attitude toward citizen participation was positively related to all four types of reported behavior, while the influence of moral emotions only related to social control and responsive behavior. These results can be used in the design and testing of interventions to stimulate citizen participation.

## 1 INTRODUCTION

In the police domain, there has been more and more attention to the large potential of citizen capital in fighting crime and creating safer neighborhoods (Bullock & Sindall, 2014; Gill, Weisburd, Telep, Vitter, & Bennett, 2014). While traditional forms of policing rely on responsive control, in which only the police are responsible for fighting crime, a more recent police philosophy called *community policing* focuses on the cooperation between the police and the community (Gill et al., 2014; Kerstholt, De Vries, & Mente, 2015). Community policing comprises three key elements: organizational transformation, problem solving, and citizen participation.

Organizations are transformed to facilitate the community policing philosophy by the alignment of management, structure, and information systems. To focus more on problem solving, the processes used by police officers are focused on proactively and systematically identifying problems and the development of effective responses. Citizen participation is about partnerships between the police and individuals and organizations in the community (COPS, 2014). So far, research on community policing has, on the one hand, focused on the effects of community policing on crime reduction and subjective safety. On the other hand, it tapped into organizational issues, such as how the police organization should be structured for the effective implementation of community policing (Connell, Miggans, & McGloin, 2008; Terpstra, 2010; Weisburd & Eck, 2004). One reason for the increased attention to the use of citizen capital in the police domain is that the police simply do not have the resources to be constantly present. At the same time, citizens know the ins and outs of their neighborhood; they know where problems lie, and when something suspicious is going on. As such, they can be widely used as the eyes and ears of the police.

To date, citizens are mostly involved in policing activities as the ‘eyes and ears’ of the police by providing intelligence (Terpstra, 2010). For example, when someone is missing, the police can send a message to a large group of citizens to look out for that person, significantly increasing the chance that the person will be signaled. One example of a citizen participation success story covered by the media in the United States was when a hospital worker spotted an abducted 4‐year old girl just after seeing an amber alert message on social media during her lunch break and notified the police, which led to her rescue (Sowers, 2016). Another Dutch example of citizen participation concerned the capture of a burglar where the police asked the public in the specific area for more information and gave them a suspect description. That same evening the police were able to make an arrest in the case after receiving multiple tips from citizens (Nu.nl, 2016).

This is not the only way citizens can be involved, however. Recently, Van der Land, Van Stokkum, and Boutellier (2014) made a classification of a broad range of citizen involvement. Categories of participation include surveillance in the neighborhood, contributing to criminal investigations by providing intelligence, conflict mediation, advising the police about the main problems in the neighborhood, seeking personal contact with the police, and collecting and spreading information amongst neighbors regarding safety. However, this classification is based on a distinction of specific tasks that can be performed by citizens. Thus, it is organized around the physical manifestation of the activities and does not necessarily depart from the perspective of citizens themselves.

However, choices whether or not to participate are arguably based on psychological drivers, which may be unrelated to physical manifestations. Moreover, attempts to influence citizens to participate with the police would require insight into these very drivers to be effective rather than their mere behavioral manifestations. For example, the specific categories of surveillance and conflict mediation in the classification proposed by Van der Land et al. (2014) may both be instantiated by the same psychological drivers, such as a concern for others. Therefore, we posit that a meaningful categorization of participation activities is preferably based on the co‐occurrence of actual participation behavior reflecting similar underlying psychological drivers of the activities.

Although the large potential of citizens in the police domain is increasingly acknowledged, research shows that, in reality, only a small proportion of citizens is actually participating (Bullock & Sindall, 2014; Van Sluis, Cachet, Van Os, Prins, & Marks, 2010). This is the case despite government programs to stimulate participation (Maton, 2008), and despite the ever‐increasing possibilities for communication between police and citizens, afforded by for example social media (Fieseler & Fleck, 2013). Given this low participation level, more studies on the psychological drivers of citizens’ participatory actions are warranted. After all, the motivation to reap the benefits of community policing sparks interest in ways to increase citizens’ participation and, hence, of the psychological drivers of participation.

In the present study, the focus will therefore be on citizen participation in the police domain and the underlying psychological mechanisms. For the scope of this study, we operationalize citizen participation in the police domain as all behaviors citizens can perform to increase safety in the neighborhood. This concerns a wide variety of behaviors (e.g., reporting crime, joining a neighborhood watch, attending a meeting with police officers) and includes both individual action and collaboration with neighbors or/and the police. To gain more insight into drivers behind citizen participation, a first aim of our study is to examine which participation behaviors co‐occur. Second, we are interested in whether they are influenced by different psychological drivers.

### 1.1 Theoretical background

#### 1.1.1 Drivers for citizen participation

Previous studies have focused on drivers of participation in the police domain. Pattavina, Byrne, and Garcia (2006) showed, for example, that social cohesion and public social control were main influencers of participation in crime prevention behaviors. Choi and Lee (2016) additionally found that citizen cooperation in community policing was influenced by community attachment, crime problems in the community, confidence in the police, and personal gains of participating. Also, police legitimacy was shown to be a driver for cooperation with the police such as reporting crime and the willingness to join meetings with the police (Tyler & Fagan, 2008). However, these drivers are mainly based on interpersonal and institutional factors and not many of these factors focused on how individual decisions to participate in the police domain are made. As an addition, we will therefore further explore which psychological drivers might influence citizens’ decision to participate in the police domain.

#### 1.1.2 Attitude

Citizens may have a broad range of opinions that are relevant for participation behavior. For example, their decisions whether to participate can be influenced by opinions about who is primarily responsible for the safety in their neighborhood or the extent to which they themselves are actually able to reduce crime. This particular set of evaluative judgments can be described as the attitude toward citizen participation. Because attitudes are generally known to be a driver for behavior (Ajzen & Fishbein, 2005; Bohner & Dickel, 2011), the attitude toward citizen participation will most likely be related to actual participation in the police domain as well. Previous research, for example, showed a relationship between attitude toward police legitimacy and the willingness to participate among youths (Hinds, 2009), and a relation between the presence of community policing programs to increase positive contact between youth and the police and attitude (Leroux & McShane, 2017). Attitudes tend to be strong and resistant to change and bias because they are often based on values or are of personal relevance (Ajzen & Fishbein, 2005; Honkanen & Verplanken, 2004).

In theories of reasoned action and planned behavior, values are one of the background factors of behavioral beliefs that are related to attitude (Ajzen & Fishbein, 2005). Research suggests that moral values are particularly important in this respect (Honkanen & Verplanken, 2004; Sparks & Shepherd, 2002). In the context of citizen participation in the police domain, for example, this might occur when citizens would regard participation in safety measures as a moral duty or when they personally experience safety problems. Hence, underlying drivers for the attitude of participation behavior appear to be of a moral nature (Boer & Fischer, 2013; Honkanen & Verplanken, 2004; Slovic & Västfjäll, 2010; Sparks & Shepherd, 2002).

#### 1.1.3 Morality in the police domain

Crime, disorder, and morality are three interwoven concepts (Cromby, Brown, Gross, Locke, & Patterson, 2010). In the police domain, citizen participation is aimed at discouraging others’ wrongful behavior, such as reducing crime and disorder, and promoting “right” behavior (Fischer & Poland, 1998). To determine what is right or wrong, people need to make a moral assessment of a situation or certain behavior based on their personal moral values. When witnessing a crime, for example, citizens assess the crime as morally wrong, which might lead them to report it to the police. More long‐term participation behaviors, such as joining a neighborhood watch or thinking along with police policy, can be instigated by moral assessment of long‐term disorder and crime in one's own neighborhood as well.

Moral values and moral emotions are comprehensively described in the moral foundations theory (Haidt, 2012). As noted above, moral values form the foundation of moral reactions, whereas emotions are instigated when these values are breached. Haidt (2012) distinguishes between five main moral values: care, fairness, loyalty, authority, and sanctity. These values are triggered by specific cues, for example, care is triggered when a person assesses that others are suffering or in distress. Alternatively, someone being robbed of his property can likely triggers values of fairness. When there are behavior conflicts with one's moral expectations and values, this likely results in the experience of emotions such as anger, shame, or gratitude.

The function of these emotions is twofold. First, they arise when a certain behavior is in line with or against a moral code and indicate to the experiencer whether the behavior should or should not be accepted. Second, these expressed emotions show violators that when they breach someone's values, they might have to adapt their behavior in the future (Harkness & Hitlin, 2014). Prior research has also shown that the adherence to moral values increases the likelihood of moral action or ethical behavior in volunteerism (which is presumably related to participation behavior; Derryberry, Mulvaney, Brooks, & Chandler, 2009). To sum up, the adherence to moral values is likely to elicit the experience of moral emotions and might subsequently lead to participation behavior. Hence, important drivers regarding morality in participation behavior in the police domain are moral values (Haidt, 2012; Honkanen & Verplanken, 2004; Sparks & Shepherd, 2002) and moral emotions (Haidt, 2003; Slovic & Västfjäll, 2010).

With regard to emotions, four families are distinguished between. Other‐condemning emotions comprise negative feelings about the character or actions of others and include anger, contempt, and disgust. Self‐conscious emotions are, from an evolutionary perspective, focused on helping oneself to fit into groups and include shame and embarrassment. Other‐praising emotions are positive emotions regarding others and contain gratitude, awe, and pride, and, finally, sympathy is mentioned as the most important other‐suffering emotion. Furthermore, some emotions are arguably associated with certain moral foundations (e.g., disgust with the sanctity foundation and sympathy with the care foundation), but so far, no framework exists that clearly integrate the moral values with moral emotions (Graham et al., 2012; Haidt, 2003; Horberg, Oveis, & Keltner, 2011).

**Figure 1:**
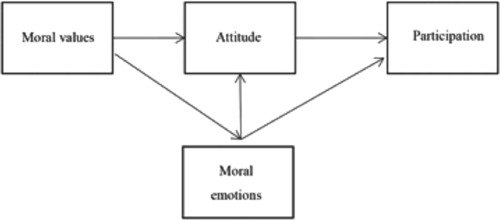
Expected model of relations between participation behavior and the attitude, moral values, and moral emotions

To summarize, we expect moral values to be the foundation for the attitude toward citizen participation and the experienced emotions. Furthermore, we expect the attitude and emotions to mediate the relation between the adherence to moral values and participation behavior, and the emotions to influence attitude. These expected interrelations are displayed in Figure 1.

### 1.2 Present study

The main objective of this study is to examine whether actual participation behavior in the police domain can be predicted by three different but connected psychological drivers: the attitude toward citizen participation, one's moral values, and moral emotions. We furthermore explore whether a classification of participation behavior in the police domain can be made and whether the influence of the psychological drivers will differ across different types of participation behavior.

## 2 METHOD

### 2.1 Participants

Data gathering was conducted in the period 2015–2016. Because we were interested in drivers of actual participatory behavior, and given the anticipated low participation rates, we contacted a wide variety of organizations and groups throughout the Netherlands in which citizens were already participating in the context of community policing (e.g., neighborhood watches, neighborhood councils of citizen representatives). We asked them via personal contact and/or email to distribute an online questionnaire among their members, with the explanation that we were interested in their opinions regarding participation in the safety domain. More respondents were generated via snowballing, and included citizens who did not engage in (much) participatory action (approximately 30% of the sample). Together, this generated sufficient intersubject variability in participation behavior.

Removal of five participants who did not fill out the online survey seriously led to a convenience sample of 217 Dutch citizens. The average age of the participants was 49 years (standard deviation [*SD*] = 16.4 years), and 51.4% of the participants were male and 48.6% female. The highest educational level completed of the respondents was as follows: 0.9% primary education, 9.5% high school, 21.7% intermediate vocational education, and 67.1% higher education. Participants lived throughout the Netherlands, with all 12 provinces represented. On average, respondents had lived in their current neighborhood for 16.8 years (*SD* = 15.00 years).

Compared to the Dutch population in 2015 (50.5% men, 49.5% women; CBS, 2015), we have a few more men and fewer women in our sample. Furthermore, the proportion of higher educated citizens in the sample is higher than in the Dutch population (67.1% in the sample vs. 31.7% in the Dutch population; Onderwijs in cijfers, 2016).

### 2.2 Procedure

Participants were asked to participate in an online survey concerning citizen participation in the police domain. After giving informed consent, participants were asked about their actual participation behavior in the police domain *in the last year*. Following this, three blocks of questions were given in random order (to avoid transgression effects between these variables). These question blocks contained questions regarding moral values, moral emotions, and the attitude concerning citizen participation in the police domain. The survey ended with some demographical questions.

### 2.3 Measures

#### 2.3.1 Dependent variable

We measured participation behavior by asking participants to what extent 21 statements applied to them concerning different participation behaviors they had actively participated in during the past year. Participants rated the answers on a 7‐point Likert scale ranging from 1 (*not at all*) to 7 (*very much*). These 21 behaviors were derived from multiple existing classifications of participation behavior as described earlier (Van der Land et al., 2014; Van Steden, Van Caem, & Boutellier, 2011) and integrated to include the full range of participation behavior (see Table 1). Their relevance to the context was also checked and confirmed in several short interviews with neighborhood police officers. Examples are as follows: “calling the police,” “being a member of a neighborhood watch,” and “correcting neighbors regarding their behavior.” Because the extent to which these behaviors can be engaged in differs (e.g., becoming a member of a neighborhood watch is a onetime behavior, while one can call the police multiple times), we standardized the participation behavior items to be able to make a more informative comparison.

**Table 1:** Factor loadings for 21 items from a self‐constructed citizen participation scale in the police domain

			Collaborative participation	Social control	Responsive participation	Detection
Items	*M*	*SD*	α = .87	α = .77	α = .72	α = .66
1. Answering questions from the police	1.93	1.46	**.54**		.47	
2. Attended a round through the neighborhood with police officer	1.70	1.46	**.78**			
3. Attending a meeting with the neighborhood police officer	2.23	1.92	**.79**			
4. Thinking along with the police about their policy	2.24	1.93	**.86**			
5. Shaping and influencing police policy	1.67	1.37	**.82**			
6. Correcting others regarding their behavior	2.54	1.72		**.51**	.46	
7. Discuss problems about crime and nuisance with neighbors	3.71	2.07	.37	**.68**		
8. Being alert for signals of inappropriate behavior	4.07	1.89		**.69**		.38
9. Being a role model for the youth	4.33	2.03		**.74**		
10. Calling the police	2.46	1.72	.48		**.50**	
11. Reporting nuisance	2.39	1.76	.39		**.54**	
12. Mediating in neighborhood conflicts	1.50	1.06	.40		**.66**	
13. Keeping track of neighborhood quarrels	2.22	1.55			**.71**	
14. Identifying offenders via the internet	1.54	1.33			.30	**.58**
15. Being member of ‘citizen net’ or amber alert	2.33	1.91				**.62**
16. Being member of a neighborhood watch	1.32	1.15				**.66**
17. Being member of a neighborhood WhatsApp group	2.31	2.26				**.75**
Eigenvalue			6.58	2.07	1.61	1.31
Percentage of variance explained			31.35	9.83	7.65N	6.24

*Note*. Means (M) and standard deviations (SD) are reported before standardization. Factor loadings < .3 are suppressed, only items loading on factors with eigenvalue >1 are shown.

To examine whether participation behavior consists of multiple factors, we conducted a principal component factor analysis with varimax rotation with the 21 types of measured participation behavior. The Kaiser‐Meyer‐Olkin measure is .84, which is above the recommended value of .5 (Field, 2013). The factor analysis, with varimax rotation, shows six underlying factors with an eigenvalue larger than 1 (see Table 1). Cronbach's alpha was computed for all factors as a lower bound estimate of reliability, with a reliability of .60 considered to be acceptable (Loewenthal, 2001). Because of low reliability scores of the fifth and sixth factor, we chose to include only the four remaining factors in subsequent analyses. These factors will be further referred to as collaborative participation (α = .87), social control (α = .77), responsive participation (α = .72), and detection (α = .66), which respectively explain 32%, 10%, 8%, and 6% of the variance (56% in total).

*Collaborative participation* involves collaboration with the police, for example, by thinking about and shaping policy, attending meetings with a neighborhood police officer, or answering questions from the police. *Social control* concerns behavior regarding others in the neighborhood, such as being a role model for the youth, being alert for signals of inappropriate behavior, and discussing problems about crime and nuisance with neighbors. *Responsive participation* concerns behavior after a transgression has occurred, such as calling the police, reporting nuisance, and keeping track of, and mediating in, neighborhood quarrels. Finally, *detection* includes detecting crime or criminals through joining neighborhood watches (physical or via WhatsApp), citizen net, or identifying offenders via the Internet. As such, this factor structure implies that participation behavior can be organized around four distinguishable components. Associated items per factor and the factor structure are shown in Table 1.

#### 2.3.2 Independent variables

We measured attitude toward citizen participation by asking participants how much they agreed with 14 opinion statements concerning participation in the police domain. Participants rated the answers on a 7‐point Likert scale ranging from 1 (*never*) to 7 (*very often*; Table 2). Because no existing scale of attitude toward citizen participation exists, a scale was constructed for our research (α = .84), based on the following four elements, which are often referred to in the literature (Clary & Snyder, 1999; Lerner & Keltner, 2000): personal values (e.g., “I should contribute to the safety of my neighborhood”); skills enhancement (e.g., “By participating, I can develop myself further”); perceived effort (e.g., “It takes too much effort to be active in my neighborhood”); and allocated responsibility (e.g., “The government is responsible for the fight against crime”).

**Table 2:** Items and descriptives of the self‐constructed scale for attitude toward citizen participation

Items	Mean	*SD*
1. The help of citizens in reducing crime is meaningful	5.81	1.29
2. Citizen participation in the identification of perpetrators is useful	5.71	1.25
3. It is fun to deploy myself for the safety of my neighborhood	4.49	1.78
4. I should contribute to the safety of my neighborhood	5.33	1.61
5. The police has to ensure the safety in my neighborhood^a^	3.09	1.51
6. It is my duty to help to make my neighborhood safer	4.95	1.58
7. Only the government is responsible for the fight against crime^a^	5.38	1.63
8. Helping in my neighborhood yields personal benefits	4.54	1.58
9. It takes too much effort to be active in my neighborhood^a^	5.00	1.65
10. By participating I can develop myself further	3.96	1.67
11. Addressing neighbors regarding their behavior will have negative consequences for me^a^	4.57	1.70
12. By helping in my neighborhood, I can extend my network	4.82	1.61
13. I think it is important to help others	5.68	1.28
14. I think everyone has to take care of him‐/herself^a^	6.00	1.34

*Note*. M = mean; SD = standard deviation.

^a^Items were reverse coded.

*Moral values* were measured by asking participants to evaluate the personal relevance of the 30 statements of the validated Moral Foundations Questionnaire (MFQ; Graham, Haidt, & Nosek, 2009; Haidt, 2012). Some statements were slightly adjusted to fit our specific (Dutch) context (e.g., “Whether or not someone's action showed love for his or her country” was changed to “Whether or not someone does good deeds for his or her neighborhood”). A factor analysis did not show a clear distribution of moral values across the five foundations known in the literature (Haidt, 2012) but displayed a scattered distribution. Separate reliability scores for three of the five moral foundations showed to be unacceptably low as well (αs below .60). Therefore, we chose to take all moral values items as one construct (α = .85) to measure the degree of adherence to moral values instead of differentiating between types of moral value dimensions.

*Moral emotions* were measured with the use of the moral emotions: anger, disgust, contempt, shame, embarrassment, guilt, compassion, gratitude, elevation, fear, pride, and schadenfreude (Haidt, 2003). Participants were asked to rate to what extent they felt these emotions in general on a 7‐point Likert scale ranging from 1 (*not at all*) to 7 (*a lot*). Because we wanted to measure actual participation behavior that has already taken place, we asked about their dispositional emotions (emotions with which people tend to react across situations and time) instead of momentary emotions (immediate affective reactions to a specific object or situation; Gambetti & Giusberti, 2009). However, previous research showed that dispositional and momentary emotions are strongly related and should yield similar results (Gambetti & Giusberti, 2009; Lerner & Keltner, 2000).

A factor analysis was executed to discover different factors, which showed three main factors. These factors–largely in line with the classification proposed by Haidt (2003)–can be labeled as *self‐conscious emotions* (guilt, embarrassment, shame, fear, and sympathy; α = .79, eigenvalue [EV] = 3.63, *R^2^* = 33.0%); *other* *‐condemning emotions* (contempt, disgust and anger, α = .71, *EV *= 1.78, *R^2^* = 16.2%); and *other‐concerning emotions* (gratitude, awe, and pride, α = .64, *EV *= 1.22, *R^2^* = 11.1%). Schadenfreude was loading on a fourth factor, but it also loaded sufficiently on other‐condemning emotions (.48). Therefore, we added schadenfreude to other‐condemning emotions (α = .70).

### 2.4 Common method bias

Because the dependent as well as the independent variables were self‐reported measures collected at the same time and with the same instrument, there is a chance on variance in the data that is attributable to the data instead of the constructs measured (Podsakoff, MacKenzie, Lee, & Podsakoff, 2003). To investigate whether the common methods approach biased the data, we conducted a Harman's single‐factor test for all four models, which all showed a common variance < 28.3%, which is under 50% and therefore seen as acceptable (Lowry & Gaskin, 2014). This suggests that the variance in the data is not likely to be attributed to the common method bias.

## 3 RESULTS

### 3.1 Descriptive statistics and correlations

Means, standard deviations, reliabilities, and correlations for the dependent and independent variables are shown in Table 3. Mean scores of all separate participation behaviors can be found in Table 1. Mean scores of the four types of participation behavior (before standardization) showed that participants are mostly involved in social control (mean [*M*] = 3.67, *SD* = 1.49), second, in responsive participation (*M* = 2.14, *SD* = 1.13), and, the least, in collaborative participation (*M* = 1.96, *SD* = 1.43) and detection (*M* = 1.88, *SD* = 1.19). This means that respondents are more likely to control behaviors of others in their neighborhood and report to the police than to actively collaborate with the police in meetings or become active themselves in detecting crimes, for example, by joining a neighborhood watch. Although the sample included relatively active citizens, the mean scores for participation behavior were still moderately low (below half‐point scale).

**Table 3:** Means, standard deviations, reliabilities, and intercorrelations among the variables

Variables	*M*	*SD*	α	1	2	3	4	5	6	7	8	9
Participation behavior												
1. Collaborative participation	1.96^a^	1.43^a^	.87	—								
2. Social control	3.67^a^	1.49^a^	.77	.49**	—							
3. Responsive participation	2.14^a^	1.13^a^	.72	.60**	.57**	—						
4. Detection	1.88^a^	1.19^a^	.66	.43**	.45**	.30**	—					
5. Attitude	4.95	.88	.84	.33**	.40**	.18**	.34**	—				
6. Moral values	5.08	.79	.78	.11	.10	.02	.02	.31**	—			
Moral emotions												
7. Other‐condemning emotions	2.88	1.05	.71	−.04	.03	.10	−.05	−.19**	.07	—		
8. Other‐concerning emotions	4.48	1.09	.64	.10	.26**	.06	.04	.14^*^	.15*	.10	—	
9. Self‐conscious emotions	4.48	.99	.79	−.09	−.12	−.08	−.12	.11	.10	.44**	.31**	—
10. Age	49.17	16.37	—	.20**	.16*	.10	−.03	.32**	.26**	−.14*	−.06	−.30**
11. Gender	—	—	—	−.22**	−.10	−.11	−.02	−.13	.16*	−.01	.10	.27**
12. Education	—	—	—	−.09	−.16*	−.04	−.03	−.10	−.28**	−.10	.03	.12
13. Years lived in the neighborhood	16.83	14.96	—	.19**	.18**	.04	.06	.19**	.17*	−.16*	−.16*	−.26**

*Note*. M = mean; SD = standard deviation.

^a^Before standardizing.

*p <.05, ** *p* <.01.

Because the frequencies of the measured participation behaviors participants could engage in differed, the participation behavior items were standardized before further analyses. The four types of participation do correlate with each other significantly, *rs > *.30, *ps *< .01. Respondents were more likely to perform all four types of participation behavior when they had a more positive attitude toward citizen participation (collaborative: *r* = .33, social control: *r* = .40, responsive: *r* = .18 and detection: *r* = .34, *ps *< .01). Moral values did not correlate significantly with participation. Of the three families of moral emotions, only other‐concerning emotions were correlated to one of the four types of participation behavior. The more respondents reported other‐concerning emotions, the more they engaged in social control (*r* = .26, *p *< .01).

### 3.2 Path analysis

**Figure 2:**
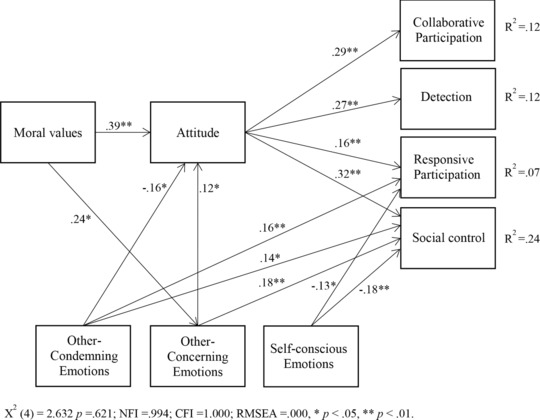
Path analysis of moral values and three types of moral emotions on four types of participation behavior in the police domain: collaborative participation, social control, responsive participation, and detection

A path analysis (with the use of AMOS) was made to examine the hierarchical relations of the constructs in an exploratory manner. We tested the expected model (Figure 1; where the three families of moral emotions were included and allowed to correlate) for the four factors of participation behavior (collaborative participation, social control, responsive participation and detection) in one model. The model showed a significant fit, X^2^(4) = 2.632, *p* = .621 (normed fit index = .994; comparative fit index = 1.000; root mean square error of approximation = .000); however, many paths showed to be nonsignificant. To illustrate the model in a clearer way, we show only the significant paths in Figure 2. Regression weights, standard errors, and significance levels of all significant and nonsignificant paths can be found in Table 4.

**Table 4:** Unstandardized regression weights, standard errors, and p values of the full path analysis model (as shown in Figure 2)

Path			Regression weight	*SE*	*p*
Moral values	→	Attitude	.39	.08	<.01
Moral values	→	Other‐concerning emotions	.24	.11	.03
Moral values	→	Other‐condemning emotions	.10	.09	.30
Moral values	→	Self‐conscious emotions	.14	.10	.15
Other‐condemning emotions	→	Attitude	−.16	.07	.02
Other‐concerning emotions	→	Attitude	.12	.05	.03
Self‐conscious emotions	→	Attitude	−.10	.07	.12
Attitude	→	Collaborative participation	.29	.06	<.01
Other‐condemning emotions	→	Collaborative participation	.05	.06	.41
Other‐concerning emotions	→	Collaborative participation	.06	.05	.24
Self‐conscious emotions	→	Collaborative participation	−.09	.06	.16
Attitude	→	Detection	.27	.05	<.01
Other‐condemning emotions	→	Detection	.05	.05	.37
Other‐concerning emotions	→	Detection	.02	.04	.68
Self‐conscious emotions	→	Detection	−.09	.05	.11
Attitude	→	Responsive participation	.16	.06	.01
Other‐condemning emotions	→	Responsive participation	.16	.06	.01
Other‐concerning emotions	→	Responsive participation	.04	.05	.36
Self‐conscious emotions	→	Responsive participation	−.13	.06	.03
Attitude	→	Social control	.32	.05	<.01
Other‐condemning emotions	→	Social control	.14	.06	.01
Other‐concerning emotions	→	Social control	.18	.05	<.01
Self‐conscious emotions	→	Social control	−.18	.06	<.01

*Note*. SE = standard error

In the path analysis, moral values were indeed located at the basis of the model and positively associated with attitude (β = .39, *p *< .01) and other‐concerning emotions (β = .24, *p *< .05). This means that when people adhere more to moral values, they had a more positive attitude toward citizen participation in the police domain and were more likely to experience emotions such as gratitude and awe. When people had a more positive attitude, they were in turn also more likely to engage in all four types of participation (βs between.16 and .32, *p*s* *< .01). Self‐conscious emotions such as guilt and shame were not related to attitude, while other‐concerning emotions (β = .12, *p *< .05) increased a positive attitude and other‐condemning emotions (β = −.16, *p *< .05) negatively influenced attitude. In other words, people who experienced emotions such as anger and disgust had a more negative attitude to participating in the police domain, while people who experience gratitude and pride more often had a more positive attitude.

When comparing the four types of participation behaviors, we see that collaborative participation and detection were influenced only by moral emotions through attitude; social control and responsive participation, on the other hand, proved to be influenced by emotions directly as well (although still as part of a mediation pathway). Respondents were more likely to perform responsive participation when they felt other‐condemning emotions such as anger and disgust (β = .16, *p *< .01) and less likely when they experienced self‐conscious emotions such as shame and fear (β = −.13, *p *< .05).

For social control, respondents were also more likely to participate when feeling more other‐condemning emotions (β = .18, *p *< .01), and less likely when they experienced self‐conscious emotions such as guilt and fear (β = −.18, *p *< .01). Furthermore, participants who experienced more other‐concerning emotions (β = .14, *p *< .01) were also more likely to engage in social control. Of the four types of participation, these models explained 24% for social control, 12 % for detection, 12% for collaborative participation, and 7% for responsive participation.

## 4 DISCUSSION

The aim of the current study was to provide insight into actual participation behavior in the context of community policing. We were interested in whether different types of participation behavior in the police domain can be distinguished based on co‐occurrence and, if so, whether these types of participation are influenced differently by three psychological drivers: attitude toward community policing, moral values, and moral emotions. The results indicate that participation behavior in the police domain should not be treated as one uniform concept, but rather should be differentiated in four different types of behavior. These are as follows, in order of prevalence: social control, responsive participation, collaborative participation, and detection. Social control involves citizens correcting each other regarding their anti‐social behavior (e.g., discussing associated problems with neighbors). Responsive participation includes behaviors as a response to crime or antisocial behavior (e.g., calling the police). Collaborative participation includes behaviors where citizens collaborate and meet with the police (e.g., answering questions from the police), and detection focuses on detecting crime or assisting in the identification of offenders (e.g., being a member of a neighborhood watch).

Because the models explain only a small proportion of variance, the results should be interpreted with caution. Our findings showed that the attitude toward citizen participation is a strong predictor for all four types of actual participation behavior, which is consistent with our expected model as well as the connection between attitude and behavior found in previous research (Ajzen & Fishbein, 2005; Honkanen & Verplanken, 2004). Attitude concerns a set of evaluative judgments toward community policing, such as whether participating is seen as meaningful in reducing crime, whether it takes too much effort, or is seen as the sole responsibility of the police and not that of citizens. An implication of our finding would be that influencing attitudes toward citizen participation can stimulate behavior. This could be done, for example, by stressing the usefulness of citizens’ participation to the police as well as to citizens themselves, or to accentuate the responsibility citizens have to participate in society.

We predicted that attitude would emanate from moral values, which we extracted from Haidt's moral foundations theory. As expected, our results did show that the adherence to moral values was indirectly associated with all four types of participation through attitude and moral emotions, although this indirect relation applied only for other‐concerning emotions. Note, however, that this concerns moral values as a general construct because we did not find support for the five separate moral foundations this theory describes (Graham et al., 2012; Haidt, 2012). The same low reliabilities of these five foundations were found in previous research with the MFQ in the Netherlands (Quintelier, Ishii, Weeden, Kurzban, & Braeckman, 2013), suggesting that the MFQ is culturally sensitive. Nevertheless, analyses with a general scale for moral values importance show that that people who consider morality more important have a more positive attitude toward community policing as well as more other‐concerning emotions (e.g., gratitude and pride).

Comparing the influence of psychological drivers on the four types of participation, we also found some apparent differences with regard to moral emotions. It stands out that collaborative participation and detection are not directly influenced by moral emotions (only through attitude), while social control and responsive behavior are (even though still as part of a mediation pathway). An explanation might be the more preventive and long‐term nature of collaborative participation (e.g., discussing crime and nuisance with the police or on a policy level) and detection (e.g., joining a neighborhood watch), compared to the more reactive and direct nature of social control (e.g., correcting others regarding their behavior) and responsive participation (e.g., reporting nuisance and mediating in neighborhood conflicts), which are more likely to be evoked by moral emotions. As results showed that social control and participation behavior were performed relatively more by participants in comparison to detection and collaboration, this might suggest that citizens are more inclined to participate when the behavior is emotionally driven.

Although this study was exploratory, these results can give policy makers some insight in participation behavior in the police domain and in ways to influence this behavior. For example, when the police want to design strategies to stimulate responsive participation, such as calling the police or reporting nuisance, they can focus on emotions. These strategies could focus on increasing feelings of disgust and anger toward the perpetrator as well as decreasing feelings of fear and guilt people might experience in anticipation of making the report. This could, for example, be done in the form of leaflets or commercials, or on a smaller scale, in communication between neighborhood police officers and their neighborhood. However, this might have ethical constraints and might lead to negative consequences such as vigilantism.

When stimulating collaborative participation, such as joining meetings with neighborhood police officers, these strategies could work more effectively when the focus is more on a positive attitude toward citizen participation and less on emotions. Further research on whether–and if so how–attitude and moral emotions can be influenced to stimulate participation behavior is recommended. In a practical setting, it would, for example, be interesting to examine further whether a neighborhood police officer can influence other‐concerning emotions in the neighborhood by promoting social cohesion. In addition, whether this in turn might lead to more social control among citizens themselves could be an area of study. This could, for instance, be achieved by introducing already active citizens to other citizens who would like to participate to form a closer network.

### 4.1 Limitations

We acknowledge some limitations of the current study. A first limitation is that a convenience sample was used, which is not necessarily a representative sample of the general Dutch population. Therefore, we cannot make firm conclusions regarding the prevalence of participation behavior based on this study. However, because our aim was to study actual participation behavior and its underlying psychological drivers, we needed a sample with a large variety in participation behavior.

Moreover, the fact that our focus was more on establishing relationships between moral emotions, attitudes, and behavior that could reasonably be expected not to differ much between individuals and groups (e.g., different age groups, rather than assessing how the general population feels about these variables), in our view renders representativeness a relatively minor issue here (for a discussion, see Kardes, 1996, and Petty & Cacioppo, 1996). Nevertheless, in future studies, it would be interesting to also focus on citizens who are not active at all, and whether these psychological drivers could also predict why citizens do not participate.

Another limitation is that we did not measure race/ethnicity, though this might affect attitudes toward the police and therefore people's willingness to participate. In this sample, this might not have significant consequences, but in other contexts in other parts of the world, this can influence police‐community relations and the willingness to participate and should definitely be taken into account in future research (Wehrman & Angelis, 2011).

Furthermore, because the separate participation behaviors differed in possible frequency and meaning (e.g., someone can become a member of a neighborhood watch once, but call the police multiple times, and how often someone calls the police also depends on circumstance), we were not able to draw firm conclusions about the frequency of separate behaviors. However, we were able to show the existence of a classification of different subtypes of behavior, which might be explained by different underlying mechanisms.

Furthermore, the disparity of different behaviors might have led to noise in the data, but we think that by standardizing the participation behaviors before analysis as well as the reasonably large sample size, we have partially reduced this noise. The participation behaviors measured contained a range of citizen activities from very effortless to more effortful participation behaviors and might not be specific to community policing only. In line with Gill et al. (2014), we see community policing as a philosophy of cooperation between citizens and the police, in which we focused on the citizens’ perspective instead of the top‐down organizational perspective (because there has been more research so far on the latter than on the previous). Therefore, we argue that it is appropriate that more police light activities were included in the scale of citizen participation in the police domain.

Another limitation is that the attitude and moral emotions were measured after participation behavior already was performed (or not performed) and might therefore have been adjusted afterward due to experiences of participating or not. One might argue that the participation behavior could also have influenced the attitude toward citizen participation and emotions instead of the other way around. Therefore, for future research, it is recommended to take into account the attitude and emotions before the decision is made to participate as well as after the participation behavior.

Finally, this study purely focused on a few individual psychological drivers, but this does not mean that we do not acknowledge the importance of other individual indicators, such as self‐efficacy and perceived behavioral control, and societal indicators, such as social cohesion, community efficacy, or community organization, as important drivers for behavior. Hence, we recommend addressing these indicators in future research concerning citizen participation in the police domain (Ajzen & Fishbein, 2005; Kerley & Benson, 2000; Xu, Fiedler, & Flaming, 2005).

### 4.2 Conclusion

This study highlights the importance of the citizen's perspective in citizen participation in the police domain. Although the results should be treated with caution, they did point to the relevance of distinguishing between actual participation behavior in the police domain and taking into account underlying psychological drivers. The results show that four types of participation behavior can be distinguished: social control, responsive participation, collaborative participation, and detection. The degree to which people adhere to moral values showed to be an important (indirect) foundation for citizens’ participation behavior regarding neighborhood safety. Furthermore, the attitude citizens have toward community policing influenced all four types of participation, while moral emotions only play an important role in social control and responsive behavior, which also happen to be the most prevalent types of participation behavior. In practice, these findings might be of assistance in designing strategies or interventions to stimulate citizen's participation behavior in the police domain.
